# Hormonal profiles of eumenorrheic women compared to hormonal intrauterine device users

**DOI:** 10.14814/phy2.70842

**Published:** 2026-03-27

**Authors:** Brooklyn M. Wiggins, Aarzo Karimi, Patricia K. Doyle‐Baker, Saied Jalal Aboodarda

**Affiliations:** ^1^ Faculty of Kinesiology University of Calgary Calgary Alberta Canada; ^2^ O'Brien Institute of Public Health University of Calgary Calgary Alberta Canada; ^3^ Alberta Children's Hospital Research Institute University of Calgary Calgary Alberta Canada

**Keywords:** estrogen, female sex hormones, hormonal contraceptives, menstrual cycle phases, progesterone

## Abstract

The hormonal intrauterine device (hIUD) induces local changes to reproductive biology (e.g., uterus, endometrium) and has some systemic physiological effects (e.g., hormones, mood, etc.). However, the systemic hormonal changes observed in women with eumenorrheic (EUM) cycles have not been thoroughly investigated in hIUD users. Therefore, this study aimed to establish the concentrations of serum estrogen (i.e., estradiol) and progesterone for hIUD users and compare those concentrations to EUM women. Sixteen hIUD users and 15 EUM women enrolled in a larger investigation had venous blood collected during two testing sessions separated by ~14 days for hIUD and in the early follicular (EFP) and mid‐luteal (MLP) phases for EUM women. The luteinizing hormone surge was determined using urinary ovulation kits, and menstrual cycles were tracked using calendar‐based counting to confirm the EUM cycles. Progesterone and estradiol enzyme‐linked immunoassays were used to analyze the serum samples. The endogenous estrogen and progesterone concentrations were similar across testing sessions in hIUD users (*p* = 0.720), whereas for EUM women, estrogen and progesterone were greater in the MLP than the EFP (*p* = 0.004). Based on these differences between estrogen and progesterone in hIUD users and EUM women, hIUD users should be treated as a unique group of females.

## 1 INTRODUCTION

The menstrual cycle (MC) is considered a vital biological rhythm for females from the age of approximately 12–51 years, primarily identified through the cyclical changes in endogenous sex hormones, estrogen and progesterone. Females experience an average of 450 MCs in their lifetime. However, intra‐ and interindividual variability exists across MCs, resulting in distinct hormone profiles (Landgren et al., 1980; Stricker et al., 2006). Several hormone phases can be identified across the 21–35 days eumenorrheic (EUM) MC (Taipale‐Mikkonen et al., 2021), (i) early follicular phase (EFP), indicated by the start of menstrual bleeding and characterized by low estrogen and progesterone; (ii) the 14–26 h prior to ovulation and the luteinizing hormone (LH) surge; (iii) positive ovulation with elevated estrogen, spanning 24–36 h; and (iv) mid‐luteal phase (MLP), characterized by higher estrogen than the EFP and progesterone >16 nmol/L (Elliott‐Sale et al., 2021).

The MC can be disrupted by exogenous hormones found in hormonal contraceptives (e.g., progestin and ethinyl estradiol found in an oral contraceptive pill [OCP]), used worldwide for a variety of purposes (e.g., prevention of pregnancy and treating reproductive conditions) (Badawy & Elnashar, 2011; Kaunitz, 2001; Schindler, 2013). The OCP has historically been the most used hormonal contraceptive method and is the most represented in the literature (Ekenros et al., 2013; Elliott et al., 2005; Giacomoni et al., 2000; Minahan et al., 2017; Myllyaho et al., 2021; Rechichi & Dawson, 2009). The synthetic hormones in the OCP enter circulation and suppress the endogenous production of estrogen and progesterone, preventing the surge of LH and other sex hormones, subsequently inhibiting ovulation (Ackerman et al., 2021; Rechichi et al., 2009). Recently, the hormonal intrauterine device (hIUD) has gained popularity with 159 million users worldwide (United Nations Department of Economic and Social Affairs, 2022). The hIUD contains a progestin called levonorgestrel, that acts as a reversible contraceptive through the daily release of progestin directly into the uterus rather than in circulation (Beltz et al., 2022). The hormones released from hIUDs to prevent pregnancy through multiple ways, including thickening the cervical mucus, causing endometrial atrophy, inducing local inflammation, and direct toxic effects on sperm (Apter et al., 2014; Moraes et al., 2016; Ortiz & Croxatto, 2007; Smith‐McCune et al., 2020). Although hIUDs are not a new technology, their effects on endogenous sex hormone fluctuations have not been well described in the literature. Indeed, the systemic hormonal changes observed in EUM cycles have not been thoroughly investigated in women with hIUDs experiencing local changes to their reproductive biology.

Selim and Hussein (2013), for example, analyzed serum estrogen and progesterone in both hIUD and copper IUD (cIUD) users (i.e., nonhormonal intrauterine) before device insertion and at 3‐, 6‐, and 12‐month post in a study on endothelial function. They found that serum estrogen concentration for hIUD users was not different from cIUD users; however, serum progesterone levels were lower for hIUD users compared to cIUD users, which aligns with the different mechanisms of interaction the devices have with the uterine environment. This study only measured hormone concentrations in the MLP (6–9 days before the expected onset of menstruation) and did not consider EUM cycles as a comparison group for hIUD users. In another study, Cabre et al. (2024) explored the endogenous estrogen and progesterone levels in phases that were expected to have low (i.e., during menses) and high (i.e., 1 week after ovulation) concentrations of endogenous hormones. Despite finding no significant difference in estrogen, they indicated that progesterone was significantly higher in the low hormone phase for hIUD users than in the EUM group. A potential limitation in this study was that estrogen and progesterone were measured using salivary tests, which, despite being a convenient method for measuring progesterone, may lack the precision for detecting low concentrations of estrogen (Rosner et al., 2013). Prior investigators have suggested using serum hormone analysis as the gold standard for assessing ovarian hormone concentrations for research purposes (Elliott‐Sale et al., 2021).

This study, therefore, aimed to describe the serum hormonal profile of hIUD users and women with EUM cycles across two phases (EFP and MLP) of the MC. We hypothesized that estrogen and progesterone would be greater in the MLP than the EFP for EUM cycles, and due to the regular release of progestin from the hIUD, estrogen and progesterone would be similar across tests for hIUD users.

## 2 MATERIALS AND METHODS

### 2.1 Participants

This research was part of a larger study investigating psychophysiological responses to fatiguing exercise performed in different phases of the MC. Institutional approval was received from the University of Calgary Conjoint Health Research Ethics Board (REB22‐0936) and was conducted according to the Declaration of Helsinki (without registration). Our sample size calculation of 16 per group with a considered drop out of 20% was based on the rate of change in progesterone concentration between groups (hIUD and EUM) from two studies: (1) Cabre et al. (2024) (effect size = 3.12), and (2) Selim and Hussein (2013) (effect size = 1.95; G*Power version 3.1).

We recruited 34 participants using convenience sampling with a 91% (31/34) completion rate (16 hIUD; 15 EUM). In two participants, serum samples were not collected due to a needle phobia and one sample was excluded because progesterone was lower than the minimum required concentration in the MLP. Participants provided written informed consent and met the following prescreening criteria: (i) no contraindications based on the Physical Activity Readiness Questionnaire (Jamnik et al., 2007); (ii) able to perform high‐intensity cycling; (iii) aged 18–40 years; and (iv) considered to have a regular MC or currently using a hIUD. Participants were excluded if there was evidence of (i) a known mood, neurological, cardiovascular, or metabolic condition deemed likely to confound the study results; (ii) a clinically diagnosed menstrual disorder (e.g., polycystic ovarian syndrome or amenorrhea); (iii) pregnancy; or (iv) having given birth within the 6 months prior to the start of the study. A regular MC was self‐reported as (i) absence of menstrual irregularities; (ii) a predictable period within 1 week; (iii) absence of unusually painful periods; and (iv) no history of hormonal contraceptives or medication use known to affect the hypothalamic–pituitary–ovarian axis for the past 6 months. Individuals with a cIUD who had regular MCs were eligible for the EUM group (*n* = 1; Liberté cIUD; 2 years of use). Those with a hIUD must have had it inserted between the past 6 months to 5 years. Participants were asked to maintain a similar diet in the 24 h before each testing session to facilitate similar energy intake between the two testing sessions.

### 2.2 Experimental design

The larger study investigated the psychophysiological responses to fatiguing exercises performed in different phases of the MC. Therefore, in this part of the study, blood draws were performed after the cycling exercise protocol to mitigate the effect of blood draws on psychological metrics collected during exercise (e.g., perceived pain and stress responses). We acknowledge that exercise may affect endogenous estrogen concentrations (Guisado‐Cuadrado et al., 2024), and to control this effect, we standardized the time of day tests were performed between phases for each participant. hIUD users' testing sessions were spaced ~14 days apart (see explanations in “Hormonal IUD Cycles”), and EUM participants visited the laboratory for one test day during the EFP (Day 2 to Day 5 of the MC) and the MLP (7 days following the day of ovulation). EUM women were females with (i) MC lengths between 21 and 35 days resulting in nine or more consecutive periods per year; (ii) evidence of ovulation via a positive urinary ovulation kit; and (iii) correct hormonal profile (outlined in the following section) (Elliott‐Sale et al., 2021).

### 2.3 Menstrual cycle phase determination

The EUM group completed calendar counting for at least 2 months prior to the onset of testing to determine their average MC length. MC length was defined as the number of days from the start of menses in one MC to the day preceding the start of menses in the subsequent MC. The phases were determined using the “three‐step method” (Schaumberg et al., 2017). Participants communicated their first day of menses to the researcher (i.e., step one) and recorded it in the Premom Ovulation Tracker app (Easy Healthcare Corp, Chicago, IL, USA). Subsequently, they used an Easy@Home LH test strip (Easy Healthcare Corp, Chicago, IL, USA; purchased via Amazon, ASIN B0BN68BSP9) each morning starting 5 days after the end of menses until a positive urinary ovulation was recorded (i.e., step two) (Mu & Fehring, 2023). Participants were instructed to perform the test at the same time every morning and to avoid the first urine of the day. They scanned the test with a cell phone camera using the synchronized app (i.e., Premom Ovulation Tracker) that visually displayed the ratio of LH levels compared to the control line throughout the MC and identified when an ovulation test was positive. The ovulation test result was shared with the researcher to confirm if the test line was darker than the control line. Luteal phase length was defined as the number of days from the day after ovulation to the day preceding the next menses. Participants provided a blood sample during each testing session, which was analyzed for serum hormone concentration (i.e., step three).

All MCs were retrospectively classified as EUM or having a menstrual disturbance. EUM participants' hormone profiles were deemed “acceptable” when a peak in progesterone concentration was observed during the MLP greater than 16 nmol/L, and estrogen concentration was higher during the MLP than the EFP. Menstrual disturbances were defined as (i) oligomenorrhea, that is, MC length greater than 35 days, but less than 90 days (Elliott‐Sale et al., 2021); (ii) anovulation, that is, no ovulation detected by the urinary ovulation test (Elliott‐Sale et al., 2021); (iii) short luteal phase, that is, luteal phase shorter than 10 days (De Souza et al., 2010); and (iv) luteal phase deficiency, that is, progesterone concentration < 16 nmol/L in the MLP (Janse de Jonge et al., 2019). If neither case was observed, participants were deemed to have menstrual disturbance(s) and excluded from further analyses. One participant was excluded due to an identified MC disturbance; all remaining MCs were classified as EUM.

### 2.4 Hormonal IUD cycles

The MC experiences of the hIUD users in this study were discussed with the researcher before starting the study. Questions discussed with the participants included: (i) do you have a predictable period? If so, how many days do you experience menses? (ii) do you experience a pattern of symptoms associated with a natural MC (i.e., cramping a few days before menses begins)? (iii) has your MC changed since getting the hIUD inserted? and (iv) is this your first hIUD? Only five out of 16 (31%) hIUD users experienced menstruation that was predictable enough to coordinate a testing session during menses. The rest of the hIUD participants had either absent menstruation (5; 31%), menses that were nearly absent (2, 12.5%), or menses that were irregular (4, 25%). Based on this unpredictability, testing sessions could not be scheduled based on menses and instead were scheduled ~14 days apart. Prior literature indicates that hIUDs may make bleeding *lighter* and can suppress ovulation in over half of hIUD cycles (Bayer HealthCare Pharmaceuticals Inc., 2024). Ovulation methods were not employed in the hIUD user group because it is unknown when ovulation would occur.

### 2.5 Blood sampling procedures

Venous blood sampling was performed during each of the two testing sessions. A 6‐mL blood sample was drawn from an antecubital vein into a silica‐coated tube by a trained phlebotomist and then left upright for 15 min to coagulate before centrifuging. Samples were centrifuged at 2500 rpm for 10 min at room temperature. Using a 500‐ to 1000‐μL pipette, the supernatant serum was separated into two aliquots (~1000 μL each) and stored at −80°C until estrogen and progesterone analyses were performed. Total concentrations of estrogen (i.e., estradiol) and progesterone were measured in duplicate using hormone‐specific enzyme‐linked immunoassay kits (Cayman Chemical, Ann Arbor, MI; Cat# 501890, 582601). All samples were analyzed using the ELISA technique with absorbance detection following the ELISA kit instructions. The sensitivity of estrogen and progesterone detection was 6 and 10 pg/mL, respectively. To calculate estrogen and progesterone levels, a standard curve was plotted using eight standards against their absorbance. Using the mean absorbance from the duplicate of each sample, the concentration of the sample was interpolated directly from the standard curve. The intra‐assay coefficients of variation were 1%–10% and 1%–12% for progesterone and estrogen, respectively.

### 2.6 Statistical analysis

Statistical analyses were computed using IBM SPSS, V. 29.0 (IBM Corp., Armonk, NY). Participant characteristics and cycle days were analyzed using independent samples *t*‐tests. Mauchly's test and Shapiro–Wilk were used to assess sphericity and normality, respectively. Greenhouse–Geisser was used when the sphericity test was violated. Mixed model ANOVA for phase (EFP and MLP) and group (EUM women and hIUD users) was utilized to examine the hormone concentration data. Bonferroni post hoc test was used when main effects were observed, and Holm–Bonferroni corrected *t*‐tests were performed when there was an interaction effect. Statistical tests were deemed significant at *p* < 0.05. Data are presented as mean ± SD throughout unless otherwise stated.

## 3 RESULTS

Table 1 shows no difference between EUM women and hIUD users for age, weight, and body fat. The EUM cycle length was 30 ± 3 days, menses length was 5 **±** 1 day, ovulation day was 18 **±** 3 days, and the luteal phase was 12 ± 2 days. The elapsed time between testing sessions for EUM women was 16 ± 9 days and there was 17 ± 10 days between testing sessions for hIUD users (*p* = 0.733).

**TABLE 1:** Participant characteristics (mean ± SD) differences by group (all, hIUD users and EUM women).

	Total (*n* = 31)	hIUD (*n* = 16)	EUM (*n* = 15)	*p‐*value
Age (years)	23.2 ± 4.4	22.8 ± 3.5	23.6 ± 5.2	0.623
Height (cm)	167.8 ± 4.2	166.0 ± 4.7	169.7 ± 2.4	0.010*
Weight (kg)	65.8 ± 9.2	67.0 ± 11.8	64.5 ± 5.4	0.459
Body fat (%)	29.1 ± 6.5	28.8 ± 6.2	29.5 ± 7.1	0.763

*Note*: *p*‐values for independent samples *t*‐tests; *statistically significant differences. %Body Fat was measured using DXA performed on a Lunar iDXA device (General Electric Healthcare, Chicago, IL, USA).

Abbreviations: EUM, eumenorrheic; hIUD, hormonal intrauterine device.

**FIGURE 1:**
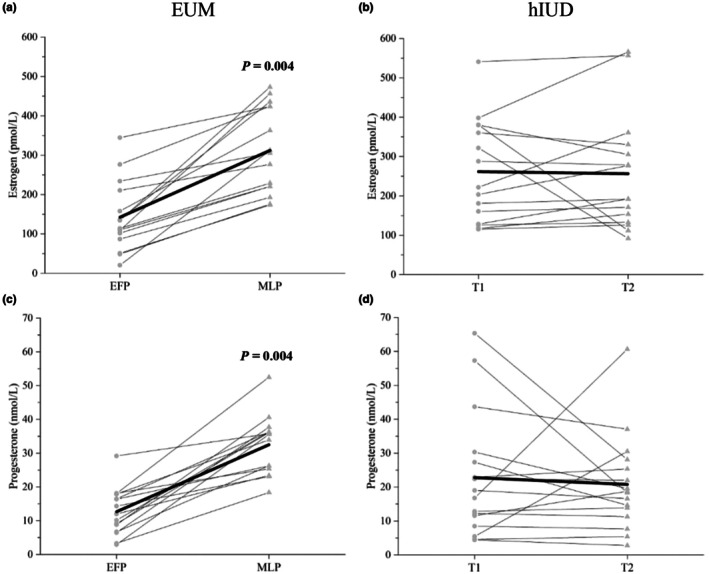
Individual concentration (light gray lines) with group mean (black line) of estrogen (pmol/L) (Panles a, c) and progesterone (nmol/L) (Panles c and d) in the early follicular phase (EFP) and the mid‐luteal phase (MLP) for eumenorrheic women (EUM; *n* = 15), and in test 1 (T1) and test 2 (T2) for hormonal intrauterine device users (hIUD; *n* = 16). Significant *p*‐values for differences in concentrations of estrogen and progesterone between the MLP and the EFP for EUM women are shown.

The mixed model ANOVA for estrogen concentration revealed a significant phase × group (*p* < 0.001) interaction effect (Figure 1), with estrogen higher in the MLP than EFP for EUM women (*p* = 0.004). T1 and T2 for hIUD users were not statistically different (*p* = 0.720).

For progesterone, a significant phase × group interaction effect was observed (*p* < 0.001), where progesterone was higher in the MLP than in the EFP for EUM women (*p* = 0.004). In T1 and T2, progesterone was similar for hIUD users (*p* = 0.703) (Figure 1).

No significant phase (*p* = 0.393), group (*p* = 0.110), or phase × group interaction effects (*p* = 0.095) were observed for the E:P ratio.

## 4 DISCUSSION

The present study explored within‐group (i.e., EFP vs. MLP) differences in endogenous estrogen and progesterone concentrations in hIUD users and EUM women. The main findings were: (i) estrogen and progesterone were higher in the MLP than EFP, in EUM women; and (ii) estrogen and progesterone were not different between T1 and T2, in hIUD users. These results demonstrate that hIUD users have unique hormonal profiles compared to women with EUM cycles, lacking the typical estrogen and progesterone variations seen in EUM women, and should be studied as a separate group.

hIUDs are traditionally understood to exert localized effects on female physiology (e.g., cervical mucus) due to direct progestin administration into the uterus (Backman, 2004; Hofmann et al., 2020; Lahteenmaki et al., 2000). Yet, evidence of systemic exposure to levonorgestrel suggests that endogenous ovarian hormones may be affected by hIUD use (Apter et al., 2014; Backman, 2004; Seeber et al., 2012). Specifically, prior studies have shown that endogenous progesterone levels in hIUD users could be different from both EUM women and cIUD users (Cabre et al., 2024; Selim & Hussein, 2013), suggesting a distinct hormonal profile. Cabre et al. (2024) examined endogenous estrogen and progesterone levels during phases that were expected to have low (i.e., during menses) and high (i.e., 1 week after ovulation) concentrations of endogenous hormones. Similar to our findings, estrogen and progesterone were not significantly different between phases in hIUD users. Their study also demonstrated significantly greater progesterone during the low hormone phase in hIUD users compared to EUM women, while no differences between groups were found in the high hormone phase (Cabre et al., 2024). As previously mentioned, their study relied on salivary hormone measurements for estrogen and progesterone, while serum hormone analysis has been recommended as the gold standard for assessing ovarian hormone concentrations for research purposes (Elliott‐Sale et al., 2021). In another study, Selim and Hussein (2013) used serum hormone analyses and found no significant differences in estrogen concentrations between hIUD and cIUD users. To clarify, the main mechanism of action of cIUDs is to prevent fertilization due to the release of copper ions into the uterus that affect sperm viability and function (Gemzell‐Danielsson & Berger, 2013). Therefore, cIUD users can have EUM cycles. These investigators found lower progesterone in the MLP for hIUD users compared to cIUD users, which was not observed in the study by Cabre et al. (2024) when hIUD users were compared to EUM women. Disparate hIUD doses and durations of use in the two studies could explain the differences between group findings for progesterone concentrations (Apter et al., 2014). Selim and Hussein (2013) only included Mirena hIUD users (52 mg) and evaluated up to 12 months of use, whereas Cabre et al. (2024) had a duration of 3.6 ± 2.3 years and did not report the hIUD dose in their sample. Based on these hormone concentration findings, it appears that progesterone levels in hIUD users do not exhibit the cyclical changes observed in EUM cycles, which could be due to systemic exposure to levonorgestrel (Apter et al., 2014; Backman, 2004; Hofmann et al., 2020; Seeber et al., 2012).

From a research design standpoint, our limited knowledge on the systemic physiological effects of hIUDs poses a foundational obstacle to increasing inclusion of female participants in research studies (Costello et al., 2014; Cowley et al., 2021). Prior literature has addressed gaps in the knowledge of how to test women in accordance with their hormonal profiles (Elliott‐Sale et al., 2021); however, the feasibility and considerations for evaluating hIUD users in basic and applied research have not been discussed to the extent that OCP users and EUM women have been. Indeed, methodological recommendations have been identified (Elliott‐Sale et al., 2021), but more are needed to appropriately define “normal” MCs for hIUD users and the hormonal time points that should be evaluated to make correct phase comparisons with EUM cycles and other hormonal contraceptive users. In addition to within‐group findings being different between groups, our findings revealed significant variations in the menstrual patterns of hIUD users that pose further complications with evaluating this group. Existing literature indicates that menstrual variations in hIUD users may depend on the progestin dose and can change over time, with more significant changes (e.g., complete absence of menses) occurring at the onset of hIUD implantation and less dramatic changes (e.g., unpredictable menses) as the device nears the end of its contraceptive effectiveness (Backman, 2004; Goldthwaite & Creinin, 2019; Lahteenmaki et al., 2000; Regidor, 2018; Xiao et al., 1995). Furthermore, previous research shows that ovulation patterns vary widely between users, ranging from suppressed ovulation in over 50% of cycles for hIUDs with higher amounts of levonorgestrel (e.g., 52 mg) (Bayer HealthCare Pharmaceuticals Inc., 2024) to minimal ovulation suppression with lower dose hIUDs (e.g., 13.5 mg) (Apter et al., 2014). Given the variability in menstrual and ovulation patterns, further research is needed to better understand the unique hormonal profiles in hIUD users. In this regard, methodological recommendations are warranted to guide researchers to effectively investigate this group of women.

### 4.1 Methodological considerations

We chose not to track ovulation in the hIUD group due to the lack of regular cycles, including a predictable period, which could be considered a limitation, even though ovulation experiences vary greatly across hIUD users (Bahamondes et al., 2020). However, without an indication of when a new cycle begins, the participant would need to take an ovulation test every day for 30–40 days. This was not possible as the study was part of a larger investigation that already had a large participant burden (e.g., uncomfortable measures for evaluating neurophysiological responses). Despite this limitation, an important strength of our study was the inclusion of qualitative insights gathered through conversations with hIUD users regarding their contraceptive experiences. This informal audit revealed substantial variability in menstruation patterns—from present, to absent, to nearly absent, to irregular—underscoring the complex physiological profiles of hIUD users. The intrauterine device, although known to have a localized response to exogenous progestin, may not always present with regular menstruation and ovulatory cycles (Goldthwaite & Creinin, 2019). These unintended but valuable qualitative findings can improve health literacy, as the process of asking questions can add to participants’ ability to understand the impact of their current choice of contraception (Nahata et al., 2023). Furthermore, these insights may help future researchers design protocols more reflective of the distinct experiences of this population. Also, blood draws in the current study were performed after the cycling exercise (see Section 2), which may raise the concern that exercise may have affected endogenous estrogen concentrations (Guisado‐Cuadrado et al., 2024). However, the literature examining the acute effects of exercise on ovarian hormone concentrations, particularly across menstrual cycle phases or contraceptive status, remains limited and controversial. For instance, several studies have indicated that moderate exercise does not affect plasma estrogen during the follicular phase (for review, see Howlett, 1987). More recently, Guisado‐Cuadrado et al. (2024) reported no significant interaction between exercise and menstrual cycle phase on hormone concentrations. Despite this evidence, Jurkowski et al. (1978) demonstrated that exercise to exhaustion could increase estrogen in the mid‐follicular phase, and both estrogen and progesterone could rise across exercise intensities during the MLP. None of the above‐mentioned works, however, has discussed the estrogen and progesterone concentrations in hIUD users. In the context of our study, exercise sessions were standardized by time of day, blood draws were collected post‐exercise in both phases, and the same exercise protocol was used in both phases. Therefore, it is unlikely that acute exercise differentially influenced hormone concentrations between phases. Accordingly, our findings remain valid and contribute novel insight into hormonal IUD profiles and highlight the need for future studies specifically designed to directly examine the interaction between exercise, menstrual cycle phase, and contraceptive use (Cabre et al., 2024).

## 5 CONCLUSION

Our findings corroborate that hIUD users do not exhibit the same fluctuations of endogenous ovarian hormones corresponding to distinct MC phases as EUM women. We acknowledge that the trend for consistent estrogen and progesterone levels with a hIUD is not new information; however, it is important to identify that these levels can vary significantly between individuals (Beltz et al., 2022). Similarly, performing an informal audit on menstruation experiences with hIUD users can inform the type of study design needed to collect best quality data. In summary, hIUD users should be treated as a unique group of females to avoid the assumption that their hormone profile mimics EUM women, given the distinctive results identified for within‐group differences in estrogen and progesterone for EUM women but not for hIUD users.

## AUTHOR CONTRIBUTIONS

**Brooklyn M. Wiggins:** Conceptualization; data curation; formal analysis; funding acquisition; investigation; methodology; project administration; resources; validation; visualization. **Aarzo Karimi:** Data curation; formal analysis; funding acquisition; investigation. **Patricia K. Doyle‐Baker:** Conceptualization; formal analysis; funding acquisition; investigation; methodology. **Saied Jalal Aboodarda:** Conceptualization; data curation; formal analysis; funding acquisition; investigation; methodology; project administration; resources; supervision; validation.

## FUNDING INFORMATION

This project was funded by the National Sciences and Engineering Research Council of Canada (NSERC) (RGPIN‐2020‐07075). B. M. Wiggins was supported by NSERC Master's and the University of Calgary Kinesiology Dean's Master's scholarships. A. Karimi was supported by the Program for Undergraduate Research Experience scholarship from the University of Calgary.

## CONFLICT OF INTEREST STATEMENT

The authors have no conflicts of interest to disclose.

## ETHICS APPROVAL

Institutional approval was received from the University of Calgary Conjoint Health Research Ethics Board (REB22‐0936) and was conducted according to the Declaration of Helsinki (without registration).

## Supporting information


**Table S1.** Brand and hIUD characteristics by years of implantation and duration (*n* = 16).

**Table S2.** Participant hormone concentrations by groups and phases.

## Data Availability

The data that support the findings of this study are available on request from the corresponding author. The data are not publicly available due to privacy or ethical restrictions.
