# Supplementary material for: Hormonal profiles of eumenorrheic women compared to hormonal intrauterine device users
Source: Physiol Rep. 2026 Mar 27;14(7):e70842. doi: 10.14814/phy2.70842 (PMC13140402; doi:10.14814/phy2.70842)
Supplement: Supplementary file 1 — Table S1. Brand and hIUD characteristics by years of implantation and duration (n = 16). [file PHY2-14-e70842-s001.docx]

**Table S1.** Brand and hIUD characteristics by years of implantation and duration (n=16).

| **hIUD**  **Brand** | **n** | **Initial**  **release rate** | **Maximum**  **duration**  **release rate** | **Years of Implantation**  **(mean ± SD)** | **Maximum duration of use** |
| --- | --- | --- | --- | --- | --- |
| Mirena | 8 | ~21 µg/day | ~7 µg/day | 1.5 ± 1.1 | 8 yrs |
| Kyleena | 8 | ~17.5 µg/day | ~7.4 µg/day | 2.7 ± 1.8 | 5 yrs |

Note: hIUD, hormonal intrauterine device.
