# Supplementary material for: Hormonal profiles of eumenorrheic women compared to hormonal intrauterine device users
Source: Physiol Rep. 2026 Mar 27;14(7):e70842. doi: 10.14814/phy2.70842 (PMC13140402; doi:10.14814/phy2.70842)
Supplement: Supplementary file 2 — Table S2. Participant hormone concentrations by groups and phases. [file PHY2-14-e70842-s002.docx]

**Table S2.** Participant hormone concentrations by groups and phases.

|  | EUM (n = 15) | | hIUD (n = 16) | | ANOVA (*P*-Value) |
| --- | --- | --- | --- | --- | --- |
|  | EFP | MLP | T1 | T2 | **P; G; P × G** |
| Estrogen (pmol/L) | 142.29 ± 89.43 | 312.05 ± 109.03^*^ | 266.13 ± 127.57 | 255.66 ± 144.29 | **< 0.001;** 0.389; **< 0.001** |
| Progesterone (nmol/L) | 12.67 ± 6.97 | 32.51 ± 8.69^*^ | 22.79 ± 18.41 | 20.78 ± 14.06 | **0.004;** 0.831; **< 0.001** |
| E/P ratio | 13.49 ± 9.00 | 10.03 ± 3.89 | 21.71 ± 23.56 | 16.51 ± 10.38 | 0.393; 0.110; 0.095 |

Note: ANOVA results show the *P*-values from the interaction effect (phase [P] × group [G]), the main effect of phase, and the main effect of group (EUM women or hIUD users). Statistically significant effects are bolded. **^*^**Greater concentration in the MLP than EFP for EUM women. EUM, eumenorrheic; hIUD, hormonal intrauterine device; MLP, mid-luteal phase; T1, test 1; EFP, early follicular phase; T2, test 2. n = 15 (EUM women) and n = 16 (hIUD users) for all variables.
